# Cynipid galls on oak leaves are resilient to leaf vein disruption

**DOI:** 10.1007/s10265-023-01462-8

**Published:** 2023-05-03

**Authors:** Marian J. Giertych, Adrian Łukowski, Piotr Karolewski

**Affiliations:** 1 Institute of Dendrology, Polish Academy of Sciences, Parkowa 5, Kórnik, 62-035 Poland; 2 Faculty of Biological Sciences, University of Zielona Góra, Szafrana 1, Zielona Góra, 65-516 Poland; 3 Faculty of Forestry and Wood Technology, Poznań University of Life Sciences, Wojska Polskiego 71c, Poznań, 60-625 Poland

**Keywords:** Gall survival, Insect–plant interaction, Oak herbivores, *Quercus petraea*, Sessile oak

## Abstract

Oaks serve as host plants for numerous insects, including those forming galls. Galls induced on oaks are completely dependent on leaf resources. Many other folivores damage veins of leaves, which may result in cutting galls off from sources of assimilates, nutrients and water. We hypothesised that the disruption of the continuity of leaf vascular tissues stops gall development, leading to the death of the larva. Leaves of sessile oak (*Quercus petraea*) with *Cynips quercusfolii* galls in the initial stage of development were marked. The diameter of the galls was measured, and the vein on which the gall was present was cut. Four experimental treatments were established: control – with no cutting, cutting the vein distal to the gall relative to the petiole, cutting the vein basal to the gall and cutting both sides. The average survival rate (live galls at the end of the experiment including healthy larvae, pupae or imagines inside) – was 28.9%. The rate varied depending on the treatment and was 13.6% in the treatment with the vein cut on both sides and about 30% in the remaining treatments. However, this difference was not statistically significant. The growth dynamics of galls are highly dependent on the experimental treatment. The largest galls grew in the control treatment, and the smallest galls were in the treatments with the veins cut on both sides. Unexpectedly, even cutting veins on both sides did not result in the immediate dieback of the galls. The results suggest that the galls are very strong nutrient and water sinks. The functions of the cut vein are likely taken over by other lower-order veins, allowing nourishment of the gall to complete larva development.

## Key message

Damage to leaf veins does not necessarily result in the dieback of leaf galls.

## Introduction

The formation of galls is one of the most sophisticated processes in the use of plants by insects (Shorthouse et al. 2005). The galling insect creates a species-specific gall through histogenetic plant changes, ensuring optimal food composition and a highly effective shelter. Galls are induced on many plant species, in particular oaks (Fagaceae), which are hosts for many gall-forming insect species, including a dozen representatives of the Cynipidae (Hymenoptera) (Redfern 2011). The mechanisms controlling gall formation are still poorly understood, and are the subject of numerous studies (Harper et al. 2004; Pawlowski et al. 2017; Schönrogge et al. 1998).

The location of gall formation initiated by oviposition is species-specific (Giertych et al. 2013; Miller and Raman 2019; Pilichowski and Giertych 2018) and determines the relationship between the gall and plant tissues (Guzicka et al. 2017; Jankiewicz et al. 2017). The vascular systems of leaves and galls are tightly integrated (Kenoyer 1936). The vascular tissues of leaves connect to gall structures and provide water and nutrients (Araujo et al. 2011; Jara-Chiquito et al. 2021; Oliveira et al. 2016). The galls of many species are large structures in relation to the leaves on which they are formed; therefore, their water and nutrient requirements are also high. Galls can act as nutrient sinks, absorbing nutrients from the leaf tissues to build the nutritive tissue of the gall (Prior and Hellmann 2010), and galls formed on leaves significantly lower the photosynthetic rates of their hosts (Larson 1998). Consequently, they are a significant burden for the host plant, especially when the occurrence is massive (Protasov et al. 2007; Stone et al. 2002).

Some galls cause an increase in the content of phenolic compounds and condensed tannins in the host plant leaves (Kot et al. 2018). Additionally, a hypersensitivity reaction creating a zone of dead cells around the galls to limit their effects has been reported in some species including oaks (Fernandes 1990; Zhang et al. 2021). Oak leaves are also frequently nibbled by other insect species, and the damage caused by herbivory can be as high as 40% (Hunter and Willmer 1989). Damage by large moth larvae (e.g. *Lymantria dispar*) often extends to the primary and secondary veins (Copolovici et al. 2017; Sohn et al. 2017). Experimental cutting of the leaf vein on *Quercus rubra* resulted in a reduction in hydraulic resistances (Sack et al. 2004). This outcome disrupts the flow of water and nutrients to the distal parts of the leaf. Venation density and transverse links may influence the mitigation effects of damage to the leaf blade in water and assimilates transport (Roth-Nebelsick et al. 2001).

**Fig. 1:**
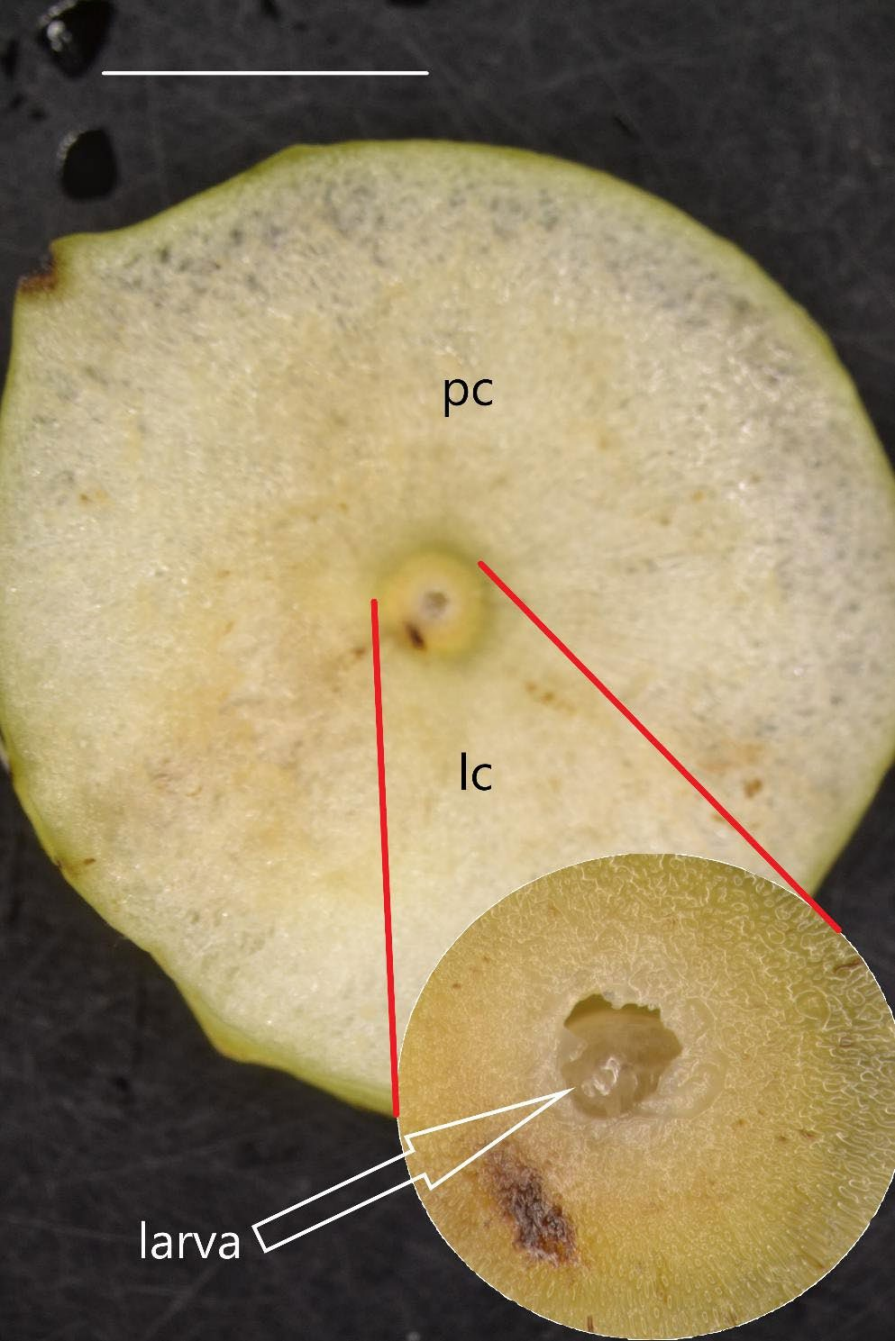
Central longitudinal section through a *Cynips quercusfolii* gall. In the centre is a larval chamber (lc) (magnified 5x in the additional photo) with visible larva (arrow) and parenchyma cells (pc). LM, Bar = 1 cm. The pictures were taken in early August after about two months of gall development

The effect of leaf damage from chewing insects on gall development has not yet been recognised. However, Schultz (1992) indicated that other insects might adversely affect gall-forming insects and suggested that damage to the leaf vein affects gall development. The current research simulates the effect of disrupting a leaf vein by chewing insects on gall development. Model objects that are an ideal fit for this type of research are galls formed by the cherry gall wasp *Cynips quercusfolii* L. (Hymenoptera, Cynipidae) (Fig. 1).

This species has a heterogonic life cycle (Pujade-Villar et al. 2001). Adults of the sexual generation emerge in spring or early summer. Females lay eggs in the new leaves after mating, then start the agamic generation, which forms large, spherical, single-chamber galls on the abaxial surface of the leaves of several oak species. Galls fall with leaves in autumn and mature on the ground. In winter or early spring, the imagines emerge from the galls and lay their eggs in the dormant oak buds, and the sexual generation develops there in little purple galls. The agamic generation starts to develop when the oak leaves are fully grown, (i.e. in June in Central Europe). The galls are attached to the primary lateral veins (sometimes to the main vein) at a constant distance from the leaf edge (Giertych et al. 2013). They are easy to find because this species primarily inhabits young oaks or lower branches of older trees (Redfern 2011). The feeding of chewing insects on oak leaves often causes damage to the leaf veins, which might disturb the integrity of the vascular tissue. We assumed that cutting the leaf vein with a razor blade could simulate the effects of the damage caused by the feeding of chewing insects. We hypothesised that integrity of the leaf vascular tissue is necessary to complete the development of the gall wasp larva, especially when the damage occurs basal to the gall.

## Materials and methods

### Study site and experimental design

**Fig. 2:**
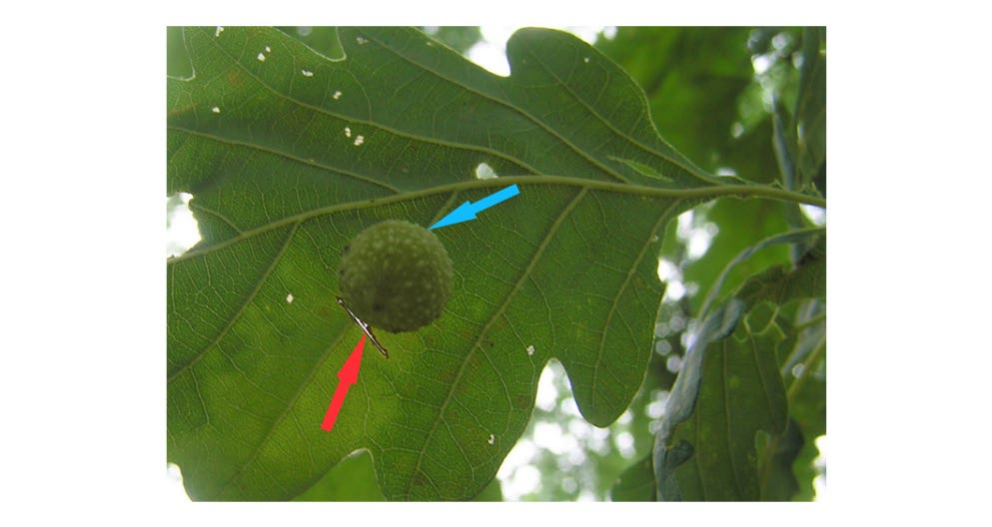
A leaf of sessile oak (*Quercus petraea*) with a *Cynips quercusfolii* gall. The red arrow indicates the crosswise cut made distal to the gall. The blue arrow marks the place of the basal to crosswise cut (not visible in this photo)

The experimental area was several hundred square metres in a typical managed mixed broadleaf forest in central Poland (52°15′53′′N; 17°00′36′′E). This study was conducted on 34 ca. 100-year-old sessile oaks (*Quercus petraea* (Matt.) Liebl) trees with low-set branches. In early July, when the galls reached about 4 mm in diameter, allowing the determination of their species, 90 young agamic-generation galls of the cherry gall wasp (*C. quercusfolii*) were identified and marked. The galls were randomly assigned to one of four treatments of artificial leaf damage. On June 5, 2011, the leaves were damaged with a razor blade by crosswise cutting the vein at a distance of 2–3 mm from the gall (Fig. 2). The selected leaves were not protected in any way against other herbivory insects or pathogens.

The control was the first group (23 replicates) without artificial leaf damage. In the second treatment (22 replicates), the leaf vein was cut 2–3 mm distal to the gall in relation to the petiole. In the third treatment (23 replicates), the cut was made 2–3 mm basal to the gall. Finally in the fourth treatment (22 replicates), the vein was cut on both sides of the gall. Before the cuts were made, the gall diameter was measured using electronic callipers with an accuracy of 0.01 mm.

In the first month of the experiment, the gall diameter was measured every other day and then weekly. The last measurement was made on September 22, when the galls were collected. The colour and firmness of the galls were noted, and their content was determined. The collected galls were classified into two categories: (1) healthy specimens (larvae, pupae or imagines) and (2) dead/unhealthy/parasitised specimens (dead larvae, parasitised larvae or imagines, damaged larvae, pupae or imagines, empty galls, etc.). Detailed data on the parasite species and the degree of parasitisation were not collected. The result for the first category within each treatment was regarded as the survival rate.

### Gall growth dynamics

The gall diameter was used to assess the dynamics of gall development during the growing season. The Richards function (Richards 1959), which is recommended for plant growth analysis (Venus and Causton 1979), was used to determine growth parameters. The goodness of fit evaluation was performed using the root mean square error. The value of the asymptote, day of asymptote achievement, and day reaching the inflexion point were calculated based on the derivatives of the Richards functions. These values, calculated independently for each healthy gall maker, were subjected to further statistical analysis. A regression analysis was performed for the data from the first measurement to the maximum value and the slope was determined, which was further analysed. For the same period, the relative growth rate (RGR) was determined according to the following formula:

RGR = lnD_max_ – lnD_0_ / t_2_ – t_1_,

where *D*_*max*_ denotes maximal diameter, *D*_*0*_ represents the starting diameter, *t*_*1*_ indicates the first day of experiment, and *t*_*2*_ represents the day of reaching the maximum diameter.

### Statistical analyses

The chi-square (χ^2^) test was used to compare the survival rate of the treatments. The analysis of covariance (ANCOVA), and Tukey’s honest significant difference (HSD) test were used to determine the difference in the Richards function parameters between treatments, with the diameter on the first day of measurement used as a covariate. The one-way analysis of variance (ANOVA) was used to determine the slope of the regression line and differences in RGR. The normality of the distribution was evaluated with the Anderson–Darling test and unequal variances were assessed using the Brown–Forsythe test. The treatment in which the leaf vein was cut on both sides was excluded from some statistical analyses because the number of galls that completed development was too small (only three specimens survived). All analyses were performed using JMP 15.0.0 software.

## Results

### Gall survival rate

**Fig. 3:**
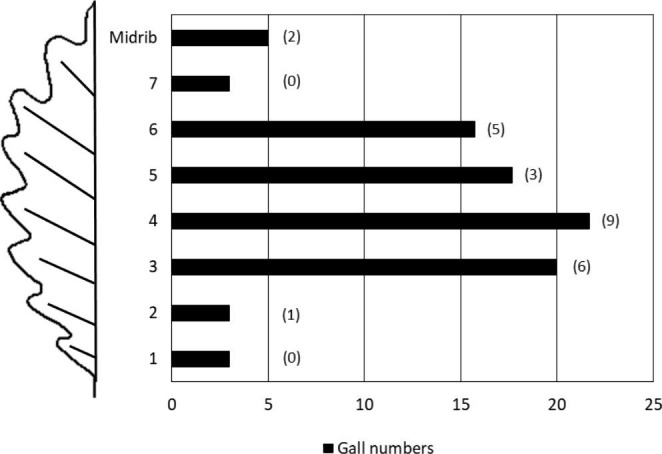
Distribution of *Cynips quercusfolii* galls on the veins of oak *Quercus petraea* leaves (*n* = 90) and the number of successfully developed galls is given in parentheses

*Cynips quercusfolii* galls were distributed on the midrib (first-order vein) or the lateral (secondary) veins (Fig. 3). The gall survival rate was not related to their location on the leaf (χ^2^ = 5,678; *p* > 0.05). Galls did not die (more precisely, the wasp larvae developing in them) after the leaf veins were cut. Most galls continued to grow, and some larvae living in them completed their development (Table 1).

**Table 1:** Initial number of galls, surviving larvae/pupae/imagines and survival rate for each leaf vein damage treatment

Vein cutting treatment	Initial number of galls	Surviving (larvae/pupae/imagines)	Survival rate (%)
Control	23	8 (2/0/6)	34.8
Distal to gall	22	7 (1/0/6)	31.8
Basal to gall	23	8 (3/1/4)	34.8
Both sides	22	3 (1/0/2)	13.6
Sum	90	26	Mean survival 28.9

The gall wasp survival rates did not differ between treatments (χ^2^ = 3,361; *p* > 0.05)

**Fig. 4:**
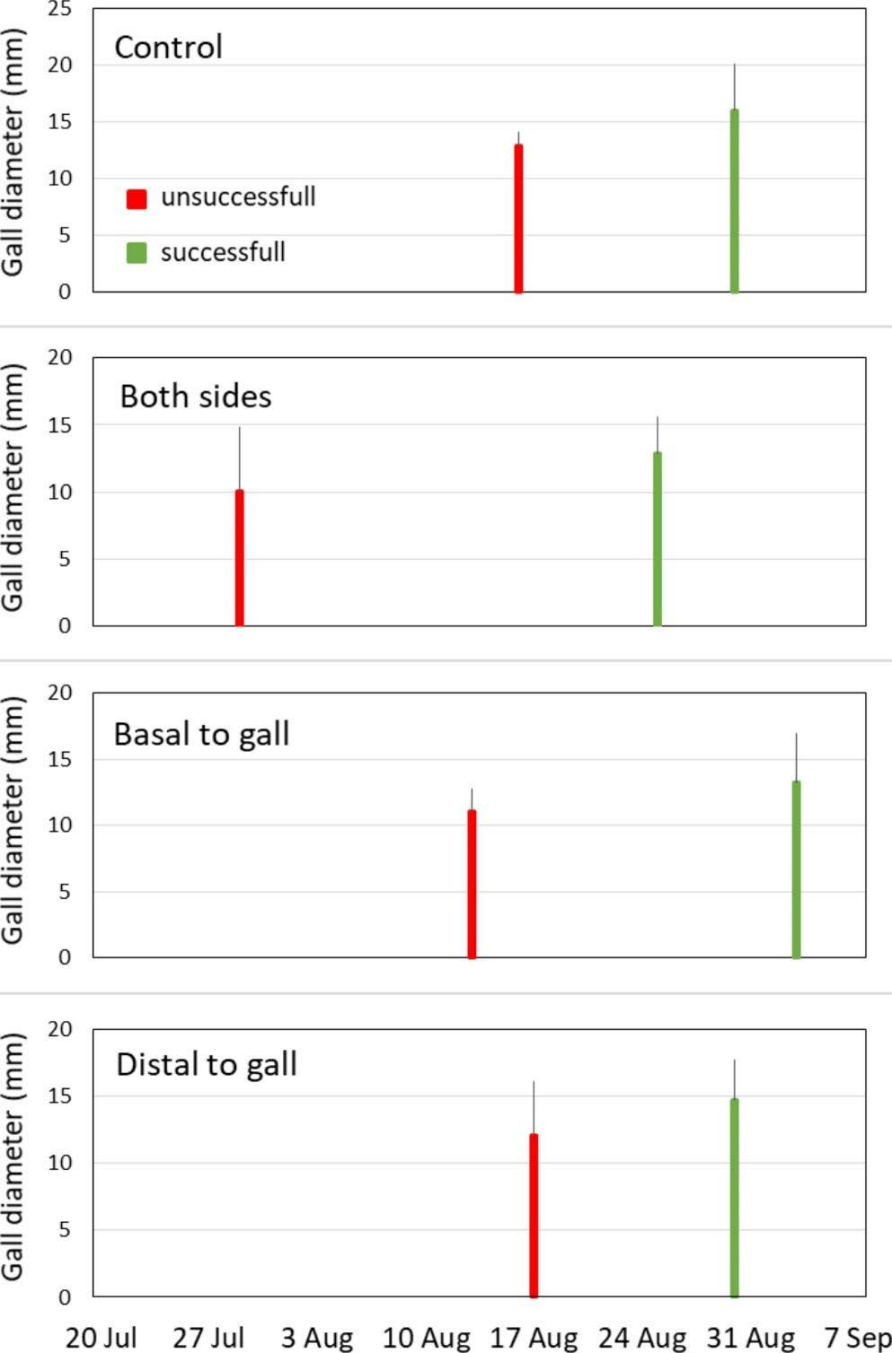
Average date of reaching the maximum diameter by *Cynips quercusfolii* galls that were successful (green bars) and unsuccessful (red bars). Error bars represent the standard error for the average maximum size

The galls, whose development was unsuccessful stopped growth about two weeks earlier, and in the case of the treatment when the vein was cut on both sides, about a month earlier (Fig. 4). The gall survival rates did not differ between the four treatments (χ^2^ = 3,361; *p* > 0.05). The development of a gall was considered successful when it contained a properly developed imago (18), pupa (1), or healthy larva (7). In contrast it was regarded as unsuccessful when it contained a damaged imago (1), parasitised imago (1), or parasitised larva (1) or was empty (7; possibly after the parasites left the chamber) or deformed without an insect inside (54; causes of gall wasp deaths were undetermined).

### Gall size

**Fig. 5:**
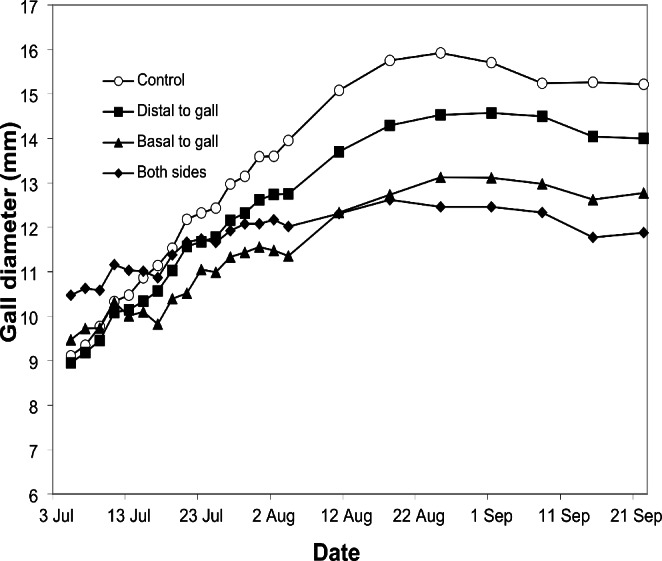
Growth curves for the mean diameter (mm) of successfully developed *Cynips quercusfolii* galls on oak *Quercus petraea* leaves for each leaf vein damage treatment (control: *n* = 8; distal to gall *n* = 7; basal to gall *n* = 8, both sides *n* = 3)

The final size reached by the galls differed significantly between the four treatments. The control galls had the largest diameter, followed by the treatments with the cut distal to the gall and the cut basal to it (Table 2). The end of growth (the day the asymptote was achieved) did not differ significantly between the treatments. (Fig. 5; Table 2).

**Table 2:** Mean maximal gall diameter, achievement of asymptote, and inflexion point; and analysis of covariance (ANCOVA) results for vein-cutting treatments with the initial diameter as the covariant for maximal diameter, asymptote achievement and inflexion point

Vein cutting treatment	Maximal diameter (mm)			Asymptote achievement (day)				Inflexion point (day)		
	Mean (SE)			Mean (SE)				Mean (SE)		
Control (*n* = 8)	15.57 (0.73) a			35.70 (2.92) a				22.67 (2.65) b		
Distal to gall (*n* = 7)	14.31 (0.78) ab			40.45 (3.12) a				32.28 (2.84) ab		
Basal to gall (*n* = 8)	12.94 (0.73) b			45.17 (2.93) a				33.82 (2.66) a		
ANCOVA	df	*F*	*p*		df	*F*	*p*	df	*F*	*p*
Treatment	2	4.73	**0.0215**		2	2.75	0.0893	2	5.15	**0.0163**
Initial diameter	1	49.818	**< 0.0001**		1	0.67	0.4205	1	0.06	0.8020
Error df	19				19			19		

*P*-values < 0.05 are indicated in bold. Means with the same letter are not significantly different; post hoc Tukey’s HSD test, *p* < 0.05

Significant differences between the treatments were found for the value of the inflexion point (the characteristic point of the growth curve) and the slope of the regression line determined for the linear section of the growth curve (Tables 2 and 3).

**Table 3:** Mean (SE) slope of the regression line, relative growth rate (RGR) and analysis of variance (ANOVA) for the influence of vein-cutting treatments on the slope of the regression line and RGR.

Vein-cutting treatment	Regression line slope			RGR		
	Mean			Mean		
Control (*n* = 8)	0.165 (0.022) a			0.0070 (0.0007) a		
Distal to gall (*n* = 7)	0.145 (0.023) ab			0.0062 (0.0007) ab		
Basal to gall (*n* = 8)	0.077 (0.022) b			0.0043 (0.0007) b		
ANOVA	df	*F*	*p*	df	*F*	*p*
Treatment	2	4,4519	**0.0252**	2	4.2688	**0.0286**
Error df	20				20	

*P*-values < 0.05 are indicated in bold. Means with the same letter are not significantly different (post-hoc Tukey’s HSD test, *p* < 0.05)

## Discussion

The leaf galls in many species form very strong nutrient sinks (Hartley 1998; Jankiewicz et al. 1970; Larson and Whitham 1991), which is necessary to ensure that the developing larvae have enough essential nutrients. Therefore, the ovipositing females must initiate the gall in the right place on the leaf (Giertych et al. 2013; Zargaran et al. 2011). Galls have unlimited access to the resources provided by the leaf until the plant begins to defend itself (e.g. through hypersensitivity reactions) (Fernandes and Negreiros 2001; Pilichowski and Giertych 2017), or the plant’s vascular tissue is damaged by other herbivores (Cunan et al. 2015). It is not known what the distribution of resources is at the level of oak leaves, but we can assume that any disturbance in leaf venation leads to increased water loss and reduced carbon dioxide uptake. One of the main causes of leaf damage is from herbivory. Oak leaves are damaged by a highly diverse insect fauna (Michalski and Mazur 2006). While feeding, some insects also damage leaf veins (e.g. larvae of the gypsy moth *Lymantria dispar* (Erebidae) or the beetle of cockchafer *Melolontha melolontha* (Scarabaeidae)), impairing the distribution of water and nutrients in the leaf.

This study indicates that *C. quercusfolii* galls can provide sufficient resources for the developing larvae, even when disconnected from the main source of water and nutrients by cutting the vein on which the gall is placed. This result is probably related to the structure of the leaf venation, which provides redundancy of transport pathways that minimise the deleterious effect of insects and other sources of damage on transport pathways (Sack and Scoffoni 2013). The sap flow in the secondary vein is lower than in the midrib, and sap can be transported laterally from neighbouring secondary veins via third-order veins (Zwieniecki et al. 2002). This situation demonstrates the strength of developing galls as nutrient sinks.

In this study, leaf veins supporting gall development were cut when the galls were about half their potential size; consequently, gall development partially slowed. Cutting the leaf veins significantly reduced the final size of the galls, especially in the ‘basal to gall’ treatment. Other parameters describing the growth curve of galls also changed. The time to reach the inflexion point indicates that the later dynamic growth stage was extended. The slope of the curve describing the linear stage of gall growth decreased significantly, and RGR decreased. All these changes resulted in a reduced gall size, which did not significantly affect larval survival. The lack of significant differences in survival may be due to the high all-cause mortality (nearly 70%), resulting in a small number of galls that completed development. The slightly extended gall development time in the treatments with damaged leaf veins was not statistically significant (Table 2), possibly indicating lower-order veins quickly took over the functions of supplying water and nutrients.

Growth reduction may also be associated with a disturbance in the activity of plant hormones, such as auxins and gibberellins (Bartlett and Connor 2014; Raman 2021). Gall size is a species-specific trait and plays a critical role because it determines the outcome of predatory and parasitic pressure (Stone and Schönrogge 2003). In some cases, smaller galls are more exposed to parasitoid pressure, and larger galls are more exposed to bird predation (Abrahamson et al. 1989; Start and Gilbert 2016; Zargaran et al. 2011). Gall mortality can be very high and, depending on the species, can reach 40% to even 80% (Eliason and Potter 2001; Wiebes-Rijks 1981; Zargaran et al. 2011). Analysis of the causes of larval mortality in galls in several tropical plant species identified hypersensitive reactions as the main cause of gall dieback (Fernandes and Negreiros 2001). In contrast, for galls of *C. quercusfolii*, Zargaran et al. (2011) found that about 20% of mortality was caused by parasites. In this study, the causes of mortality were not determined, but the overall mortality is consistent with other studies, and no significant differences were found in survival between treatments.

## Conclusion

The survival rate of *C. quercusfolii* was low, and the differences between the treatments were small and statistically nonsignificant. This study did not confirm the first part of the hypothesis that cutting the leaf vein basal to the gall would result in the death of the developing larvae, because, in a few cases, the larvae could complete development, and even cutting the leaf vein on both sides did not cause dieback. This outcome indicates that the gall acts as an effective sink on the leaf and that alternative pathways transport nutrients and water to the gall. This phenomenon requires further research, especially on the direction of assimilate and nutrient flows from the leaf to the developing gall.

## Data Availability

Data are available on request from authors.
